# Cellulose effects on morphology and elasticity of *Vibrio fischeri* biofilms

**DOI:** 10.1038/s41522-016-0001-2

**Published:** 2016-11-03

**Authors:** Christopher Ziemba, Yael Shabtai, Maria Piatkovsky, Moshe Herzberg

**Affiliations:** 1 Department of Desalination and Water Treatment, Zuckerberg Institute for Water Research, Jacob Blaustein Institutes for Desert Research, Ben-Gurion University of the Negev, Sede Boqer Campus, 84990 Israel; 2 Present address: Eawag, Swiss Federal Institute of Aquatic Science and Technology, Überlandstrasse 133, CH-8600 Dübendorf, Switzerland

## Abstract

Cellulose effects on *Vibrio fischeri* biofilm morphology were tested for the wild-type and two of its isogenic mutants that either exhibit increased cellulose production or do not produce cellulose at all. Confocal laser scanning microscopy imaging of each biofilm revealed that total sessile volume increases with cellulose expression, but the size of colonies formed with cellulose was smaller, creating a more diffuse biofilm. These morphological differences were not attributed to variations in bacterial deposition, extracellular polymeric substances affinity to the surface or bacterial growth. A positive correlation was found between cellulose expression, Young’s (elastic) modulus of the biofilm analyzed with atomic force microscope and shear modulus of the related extracellular polymeric substances layers analyzed with quartz crystal microbalance with dissipation monitoring. Cellulose production also correlated positively with concentrations of extracellular DNA. A significant negative correlation was observed between cellulose expression and rates of diffusion through the extracellular polymeric substances. The difference observed in biofilm morphology is suggested as a combined result of cellulose and likely extracellular DNA (i) increasing biofilm Young’s modulus, making shear removal more difficult, and (ii) decreased diffusion rate of nutrients and wastes into and out of the biofilm, which effectively limits colony size.

## Bacteriology: Cellulose production by bacteria affects their growth patterns

Different strains of the same bacterium show different sessile growth characteristics according to the amount of cellulose they produce. A team at Israel’s Ben-Gurion University of the Negev, headed by Moshe Herzberg, examined the growth of three strains of Vibrio fischeri (a bacterium found globally in marine environments) that produce different amounts of cellulose. The team found that cellulose production significantly affected the morphology of the resultant biofilm (bacteria sticking to one another and adhering to a surface). Increased cellulose production elevated the amount of extracellular DNA in the biofilm and reduced the size of the colony. The authors conclude that the morphological changes were due to cellulose and extracellular DNA increasing the biofilm’s stiffness and hampering the diffusion of nutrients and wastes into and out of the biofilm, thereby limiting the colony size.

## Introduction

Bacteria in aquatic environments exhibit a strong preference toward living in a sessile phase, attaching to a surface and developing a biofilm community.^1,2^ Living in a biofilm enables horizontal gene transfer and increases resistance to antibiotics, dehydration, changes in temperature, pH, and other environmental hazards.^1,2^ Maximizing these protections and growth opportunities while allowing sufficient exchange of nutrients and waste into and out of the biofilm requires a complex three-dimensional structure, which is held together by a matrix of extracellular polymeric substances (EPS). This EPS governs the physical characteristics of the biofilm, like strength, elasticity, and permeability. EPS on the surface of a bacterium can also contribute to initial development of the biofilm by impacting the deposition characteristics such as charge and hydrophobicity, or acting as a mechanical intermediary in attachment.^3–5^ Each of these functions and influences can have a tremendous impact on the morphology of the biofilm. Understanding which factors influence biofilm morphology and performance characteristics can improve efficiency or abilities of engineered systems.

EPS is a diverse collection of carbohydrates, proteins, lipids, nucleic acids, and other hetero-polymers produced by bacteria, which can account for up to 90 % of a biofilm’s mass.^3,6,7^ The specific components of EPS vary between different bacteria, and under different environmental conditions.^8^ Understanding the role of EPS in a biofilm requires first investigating what EPS components are present and identifying their functions on an individual basis.

This study targets cellulose, a known component of EPS in many bacteria such as *Escherichia coli*, *Salmonella spp*., *Acetobacter spp*., *Rhizobium spp*., *Vibrio spp*.^9–12^ Bacterial cellulose is a long polysaccharide chain of glucose units connected with (1 → 4) *β*-glycosidic bonds, free from side chains, which are found in lignin, pectin, and arabinan.^13,14^ Strong hydrogen bonding between hydrophilic cellulose molecules aligns individual cellulose chains into long parallel assemblies, which then form highly networked web-like structures.^13,14^ The backbone of *β*-glycosidic bonds imparts the cellulose molecule high crystallinity and considerable rigidity.^14,15^ In previous studies, bacterial cellulose has been extracted, purified, and subjected to mechanical testing.^16,17^ The isolated cellulose displayed high tensile strength, almost equal to that of aramid fibers.^16^ In the presence of water, extracted cellulose forms a hydrogel^14,18^ capable of holding a tremendous amount of water (up to 200 times of its dry mass) due to its high surface area and abundance of hydrogen-bonding sites.^17^ How cellulose behaves within the EPS matrix and how this interaction affects the mechanical properties and resulting morphology of the biofilm, according to our knowledge, have never been shown and are not well understood.

Previous studies have analyzed the impacts of cellulose on biofilm formation using mutant strains that lack the genes necessary to produce cellulose.^11,19,20^ No cellulose biofilms grown on stationary air–liquid or stationary solid–liquid interfaces have been qualitatively observed to have greater difficulty in forming, and then exhibited less cohesive strength relative to wild-type biofilms.^11,19,20^ Jonas et al. discovered very similar morphologies between no cellulose and wild-type *Salmonella typhimurium* biofilms grown on mica submerged in liquid media.^19^ In each of these studies, however,^11,19,20^ the biofilms were not grown under shear flow, which would better represent engineered applications, such as water treatment or a variety of medical devices. In the presence of shear flow, the contributions of rigidity and the cohesive strength of the cellulose may have greater discerning influences on biofilm morphology.

The implications of cellulose expression on biofilm morphology have been investigated in this study using three strains of *Vibrio fischeri*, a wild-type, an isogenic mutant that produces more cellulose, and an isogenic mutant that does not produce cellulose. Biofilms of each strain have been grown under low-shear, rich-media conditions, stained with live/dead fluorescent markers, and visualized using confocal laser scanning microscopy (CLSM). These images show that increasing cellulose expression increases total biofilm volume, but creates a biofilm morphology of smaller macro-colonies and more diffuse structure. Targeted experiments determined these differences in biofilm morphology to be most significantly linked with changes in biofilm elasticity (Young’s modulus) and changes in the rates of diffusion through the biofilm. The differences between cellulose expression strains in terms of cellular deposition, cellular hydrophobicity, cellular surface charge, adherence of the EPS matrix, and growth rates have also been investigated and their impacts on the resulting biofilm morphology are systemically discussed.

## Results and discussion

### Cellulose creates a more diffuse biofilm with smaller colonies

**Fig. 1:**
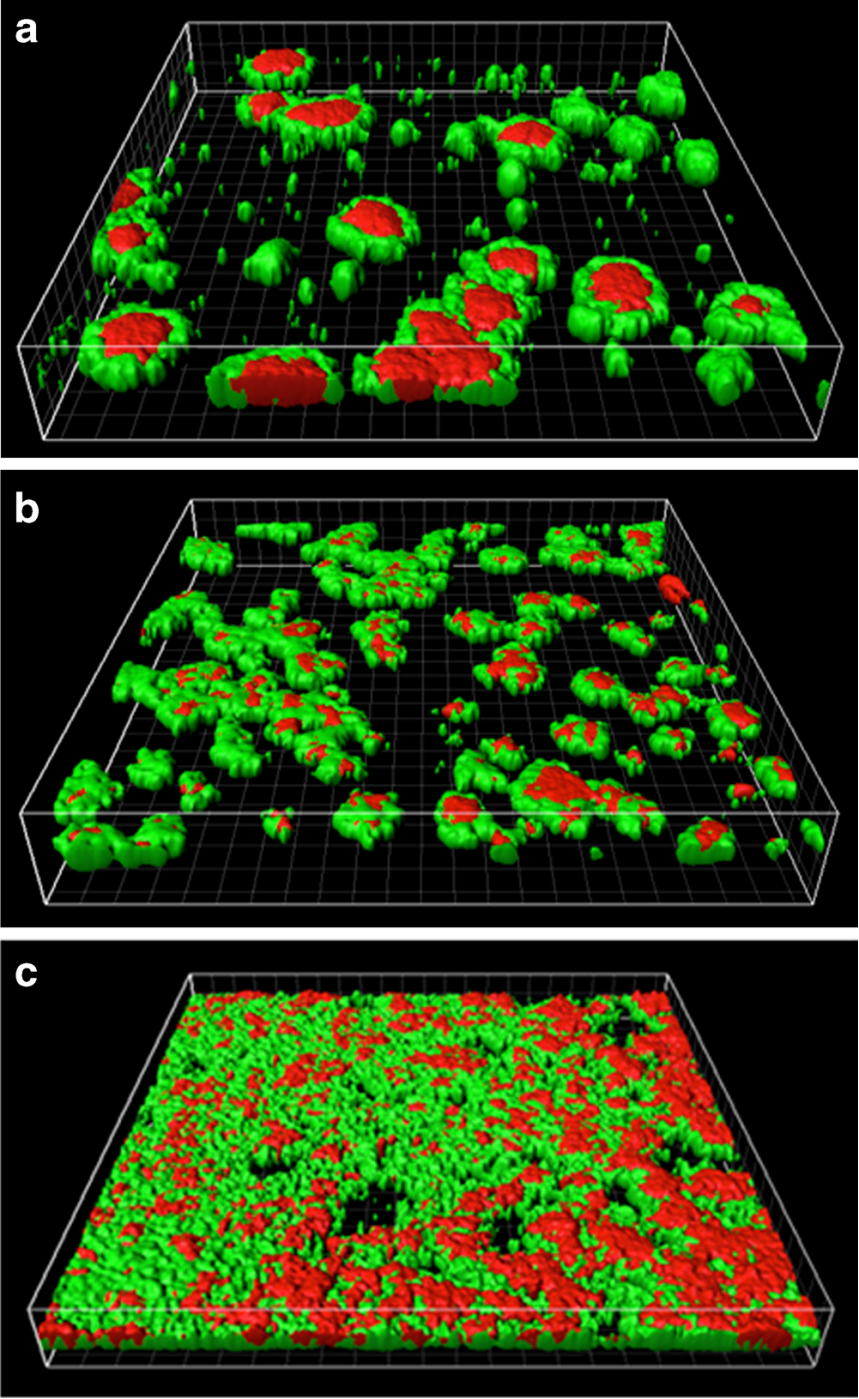
CLSM images of different biofilms formed by cellulose variants of *V. fischeri*: **a** no cellulose, **b** wild-type, and **c** increased cellulose. The *green* and *red* spots represents regions with relatively greater concentrations of live or dead cells, respectively. The figures are perspective views of a 450 × 450 µm membrane surface

The distinctive morphologies of *V. fischeri* biofilms expressing cellulose to different degrees (wild-type, no cellulose, and increased cellulose strains) are evident in representative images visualized with the CLSM and provided in Fig. 1. This figure depicts biofilms grown on polypropylene membranes over 24 h, under constant flow of rich Luria-Bertani Salt (LBS) media at 25 °C. The green stain (SYTO-9) represents areas of the biofilm that contain relatively higher concentrations of live cells, while the red [propidium iodide (PI)] represents areas with relatively higher concentrations of dead cells. In these three-dimensional images, the wild-type biofilm display approximately 75 colonies, with diameters of approximately 20–40 µm (~3.7 × 10^4^ colonies/cm^2^). We see a higher concentration of dead cells at the centers of these colonies, which may be associated with initial colony development. The healthier cells surrounding these colony centers may represent more recent growth.

**Fig. 2:**
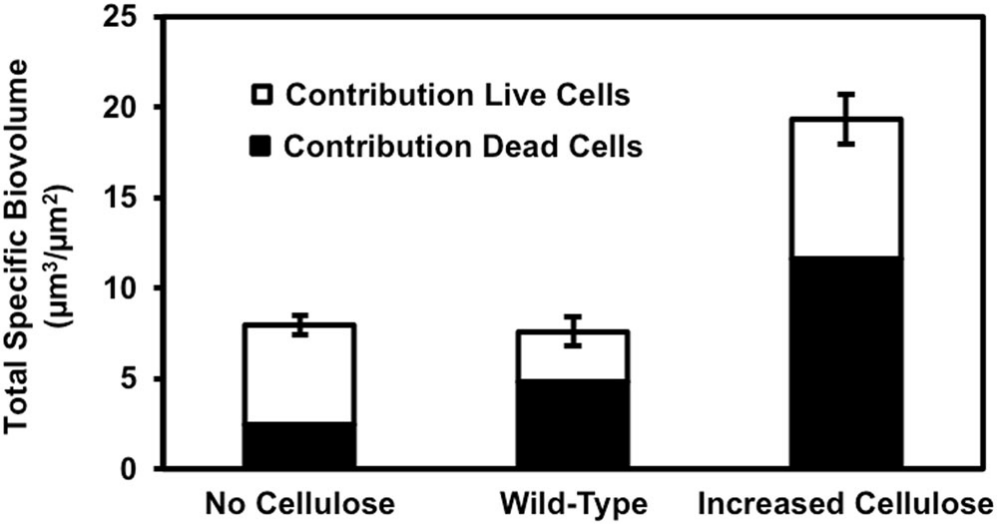
Total specific biovolumes of the biofilms formed by cellulose variants of *V. fischeri*. Each bar is a stacked total of the individual biovolume contributions from live and dead cells, identified using SYTO-9 and PI stains, respectively. Error bars represent 1 standard error

The biofilm with no cellulose is dominated by fewer colonies (approximately 21 colonies that correspond to ~1.04 × 10^4^ colonies/cm^2^), much larger (approximately 75 µm) in diameter, which also display areas of significantly more dead cells in the center of each colony. The increased cellulose biofilm exhibited a complex textured structure that nearly covered the entire membrane surface. While the total biovolumes (combining both live and dead volumes of the biofilm) are virtually identical between the wild-type and the no cellulose strains (±5 %), the increased cellulose strain produces a biovolume approximately 2.5 times higher (Fig. 2). Structurally, the increased cellulose biofilm resembles a combination of many small colonies, 5–10 µm in diameter, and larger colonies, similar in size to what we see in the wild-type. We do not see evidence of the still-larger 75 µm structures in the increased cellulose strain, which are present in the no cellulose strain. While the wild-type and no cellulose biofilms display round colony structures and seem to have grown from clear points of origination, the increased surface coverage and convergence of colonies in the increased cellulose biofilm makes it difficult to distinguish individual colonies. It is clear, however, that the number of individual colonies that successfully developed on the membrane surface increases as cellulose expression is elevated between all three cellulose expression strains. While the intrinsic variability of staining and CLSM image processing makes it difficult to draw quantitative conclusions on biofilm cells viability, the results in Fig. 2 indicate that the two biofilms containing cellulose exhibit higher ratios of live to dead cells than observed in the biofilm without cellulose.

**Fig. 3:**
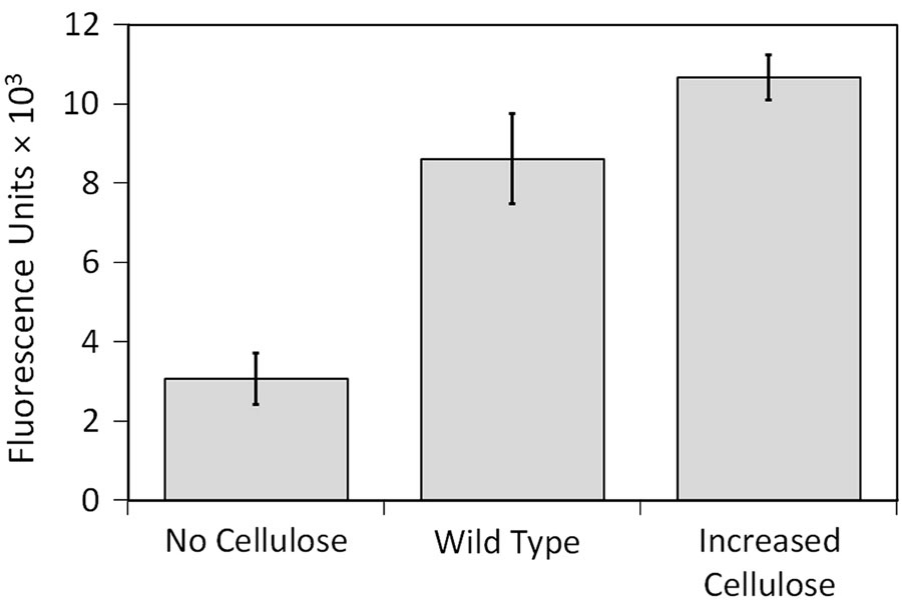
Fluorescence intensity assay with calcofluor binding to EPS extracted from biofilms of the different cellulose expression variants (excitation and emission at 355 and 434 nm, respectively)

While we believe the cellulose is the dominant EPS component influencing the different morphologies we see between our different strains, we must also consider the influence of extracellular DNA (eDNA). It is well established that eDNA is another important structural component of the biofilm matrix that can affect biofilm architecture and cohesion.^21–24^ The EPS extracted from our three *V. fischeri* strains displays increasing eDNA concentrations with increasing cellulose production. In the strain without cellulose, 8.2 ± 0.4 (SE) µg eDNA/mg total organic carbon (TOC) of EPS was detected, while for the wild-type and increased cellulose strains significantly higher amounts of eDNA were detected, 20.8 ± 0.5 (SE) and 50.6 ± 1.4 (SE) µg eDNA/mg TOC of EPS, respectively. It is then difficult to isolate the morphological effects of cellulose from the effects of eDNA. The mutations employed in this study to increase and prevent cellulose production may have additional impacts on the biofilm that are not known. The Δ*binA* mutation, which increases cellulose production, has also been shown to increase concentrations of the intracellular signaling molecule cyclic diguanylate (c-di-GMP), which is associated with biofilm formation. While the functional outputs regulated by c-di-GMP that relate to biofilm formation of *V. fischeri* are still unknown, we tried to compare the amount of cellulose in the different biofilms. A staining effort of the cellulose in the biofilms using calcofluor fluorescent stain provided a low fluorescent signal, which could not be used for comparison between the different strains. Therefore, using calcofluor, we measured the relative amount of cellulose in the EPS extracted from biofilms of the different cellulose variants. Hence, significant differences in the calcofluor binding to the different types of EPS were detected (Fig. 3) and the expected differences in cellulose production were validated: the lowest fluorescence intensity was observed for the EPS extracted from the Δ*bcsA* mutant (no cellulose), and the highest fluorescence was observed for the EPS extracted from the Δ*binA* mutation (increased cellulose). The fluorescence of the EPS with no cellulose could be attributed to non-specific interaction with other *β*-1,3 and *β*-1,4 polysaccharides. Figures 2 and 3 show that the total amount of biofilm (specific biovolume) developed for the three different cellulose variants (Fig. 2) is correlated to the amount of cellulose detected in the EPS (Fig. 3) only for the two extreme cases (no cellulose and increased cellulose). These results are not surprising as cellulose is probably not the main component of *V. fischeri* biofilms that were reported to consist mainly of the non-characterized symbiosis polysaccharide (Syp).^10^ Interestingly, even though cellulose and eDNA are probably not the main EPS components, their effect on biofilm architecture is significant. Adding exogenous bacterial cellulose to the biofilms tested could complement the phenotypes observed in the strain with reduced amount of cellulose. However, such addition would compose exogenous material with lower degree of polymerization (due to the relative low solubility of cellulose)^25^ compared to the presence of cellulose differentially synthesized in the biofilms being tested.

Our imaging of no cellulose, wild-type, and increased cellulose strains indicated dramatic changes in biofilm morphology at three levels of cellulose expression; however, this testing was conducted under a specific set of environmental conditions. A similar study investigating *Salmonella typhimurium* biofilms with and without cellulose, grown in stationary liquid media, did not display significantly different morphologies with different cellulose expression.^19^ Though our study has utilized a different bacteria (*Vibrio spp.* vs. *Salmonella spp*.), we believe the presence of shear forces in our study (vs. no shear in Jonas et al.^19^) may contribute significantly to the differentiation that we see as a function of cellulose production.

### Cellulose expression does not affect cell deposition, cell hydrophobicity, surface charge, EPS adherence, or cellular growth rates

**Fig. 4:**
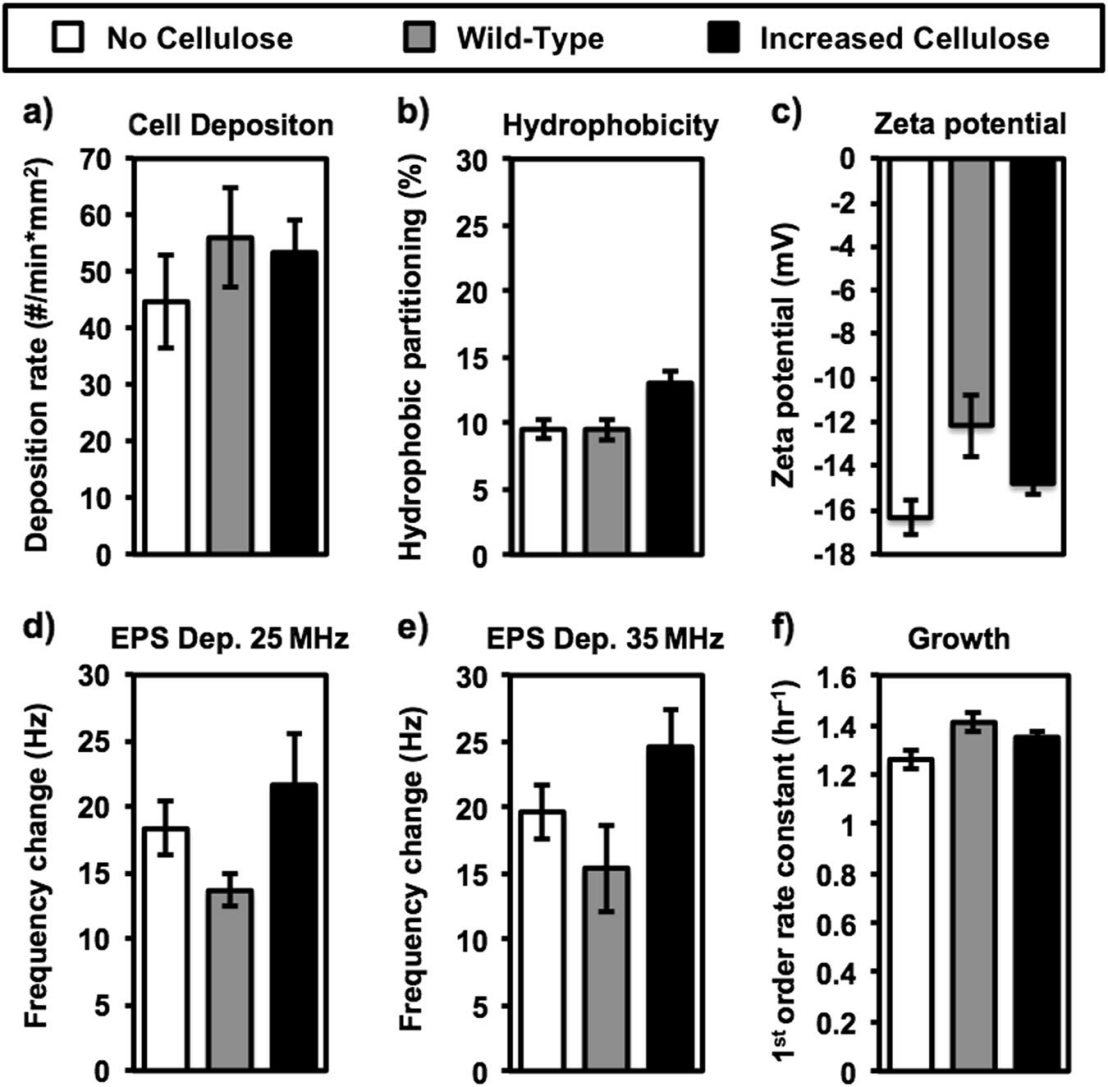
Cell deposition rate (**a**); Hydrophobic partitioning (**b**); Zeta potential (**c**); QCM-D frequency change due to EPS adherence at 25 MHz (**d**); QCM-D frequency change due to EPS adherence at 35 MHz (**e**); and growth rates (**f**), for no cellulose, wild-type, and increased cellulose strains. Bacterial cell deposition was measured directly on a polypropylene membrane using a microscope-mounted flow cell in LBS media. Hydrophobic partitioning is based on relative affinity to *n*-dodecane of planktonic cells from cell suspension in 150 mM NaCl solution. Zeta potential was measured in 150 mM NaCl solution with OD (600 nm) of 0.1. Frequency change of QCM-D sensor is presented after 1 h exposure of 12.5 mg/l as TOC of extracted EPS in 0.34 M NaCl solution for sensor frequencies at either 25 MHz or 35 MHz. Cell growth was observed in LBS media by spectrophotometer at 25 °C. All error bars represent 1 standard error

In order to estimate if changes in biofilm growth were attributed to the initial amount of attached bacteria on the surface, the initial irreversible attachment of bacteria to a similar surface was studied. Bacterial cell deposition rates were conducted under conditions that promote bacterial deposition within a comparable time period to the bacterial inoculation period applied for biofilm growth experiments. The deposition experiments were measured for no cellulose, wild-type, and increased cellulose strains to determine potential impacts on initial biofilm development and the resulting morphology (Fig. 4a). The deposition rates for each strain were measured directly on a polypropylene membrane mounted in a parallel plate flow cell used in our previous study.^26^ The no cellulose, wild-type, and increased cellulose strains displayed deposition rates of 45 ± 8.2, 56 ± 8.7, and 53 ± 5.7 (SE) cells per minute per surface area (mm^2^), respectively. There was no significant difference (*p* > 0.35) observed between these rates. Deposition results are presented in the Supplementary information (Figs. S8–S10). While increased cellular deposition rates are not always indicative of increased biofilm development,^27^ the similarity of these observed rates indicates that deposition is not responsible for the differences we see in biofilm morphology. Notably, in contrast to previous studies, in this study the higher amount of eDNA in the EPS matrix had no effect on the bacterial attachment.^21,28^ Possible reasons may include the following: (i) low amounts of eDNA at the initial cell attachment stage, or (ii) the attachment experiments contained LBS media, an LB medium supplemented with 20 g/l (0.34 M) of NaCl that likely shields any type of possible electrostatic repulsion forces between the cells and the surface, and provides favorable cell–surface interaction, even in the absence of eDNA.^29,30^

Since, both bacterial cell hydrophobicity and charge (deduced from analysis of cells’ zeta potential) have a strong impact on deposition rate,^31,32^ the deposition characteristics of each strain were further investigated by measuring partitioning from the aqueous phase onto a hydrophobic surface of *n*-dodecane as well as by measurements of the cells’ zeta potential (Figs. 4b, c). The hydrophobicity test roughly defines bacterial hydrophobicity by the following solid phase partitioning percentage (pp%) ranges: pp% > 70 % is hydrophobic, 70 % < pp% > 30 % is slightly hydrophobic, and pp% > 30 % is hydrophilic.^33^ The pp% for the no cellulose and wild-type strains are the same (9.5 %, ±0.75, ±0.81 SE, respectively, *p* = 1), while the increased cellulose strain (13 ± 0.98 % SE) is significantly different but still in the hydrophilic range (*p* = 0.024–0.026). The increase in hydrophobicity for the increased cellulose strain is contrary to our expected result, as cellulose is known to be hydrophilic.^13,14^ With a wider focus, however, we can say that each of these strains is clearly in the test’s hydrophilic range, and that this small difference in hydrophobicity does not seem to have a discerning effect on deposition rate. Analysis of surface zeta potential (Fig. 4c) reveals that each cell is negatively charged, ranging from −16 ± 0.76 (SE) mV for the no cellulose strain to −12 ± 1.6 (SE) mV for the wild-type, and −15 ± 0.33 (SE) mV with no significant differences between stains (*p* ≥ 0.23).

EPS adhesion assays were conducted in quartz crystal microbalance with dissipation (QCM-D) to determine possible effects of cellulose expression in the biofilm on the interaction between the surface and the biofilm matrix as a possible reason for different observed morphologies. The QCM-D monitors changes in oscillation frequency and dissipation character for overtones of the 5 MHz fundamental sensor resonance frequency. The data in Figs. 4d and e represent the change in overtone resonance frequencies attributed to the deposition of EPS on the sensor surface at the sensor overtone frequencies 25 and 35 MHz, which correspond to the 5th and 7th overtones, respectively. At the 5th overtone, the frequency decreases 18.3 ± 2.0, 13.7 ± 1.3, and 21.7 ± 3.8 (SE) Hz, for EPS deposition from the no cellulose, wild-type, and increased cellulose biofilms, respectively (Fig. 4d). There is no significant difference between these values (*p* > 0.1). The frequency decreases for the 7th overtone represent a similar, and also not significantly different behavior (*p* > 0.08) with frequency changes of 19.6 ± 2.1, 15.4 ± 3.3, and 24.6 ± 2.9 (SE) Hz for EPS deposition from the no cellulose, wild-type, and increased cellulose biofilms, respectively (Fig. 4e). These QCM-D deposition data illustrate that the affinity of the EPS to the polyamide-coated sensor surface is the same for each cellulose-expression mutant, and therefore does not contribute to changes in biofilm morphology. In a similar manner as bacterial attachment experiments, also in the case of EPS adsorption, eDNA had no effects on the affinity between EPS and the surface of the QCM-D sensor.

Exponential growth rates do not show a clear trend with varying cellulose expression (Fig. 4f). The growth rate constants for the no cellulose strain, *k* = 1.26 ± 0.037 (SE) h^−1^, the wild-type strain, *k* = 1.41 ± 0.040 (SE) h^−1^, and the increased cellulose strain, *k* = 1.35 ± 0.019 (SE) h^−1^, are similar, with only a slightly significant difference between the no cellulose and wild-type strains (*p* = 0.047). It is difficult to draw a conclusion from this data because we could expect that overproduction of cellulose might cause a fitness loss, as it would cost resources, but the overproduction strain is not significantly different from the no cellulose strain (*p* = 0.055).

### Cellulose affects diffusion and biofilm viability

**Fig. 5:**
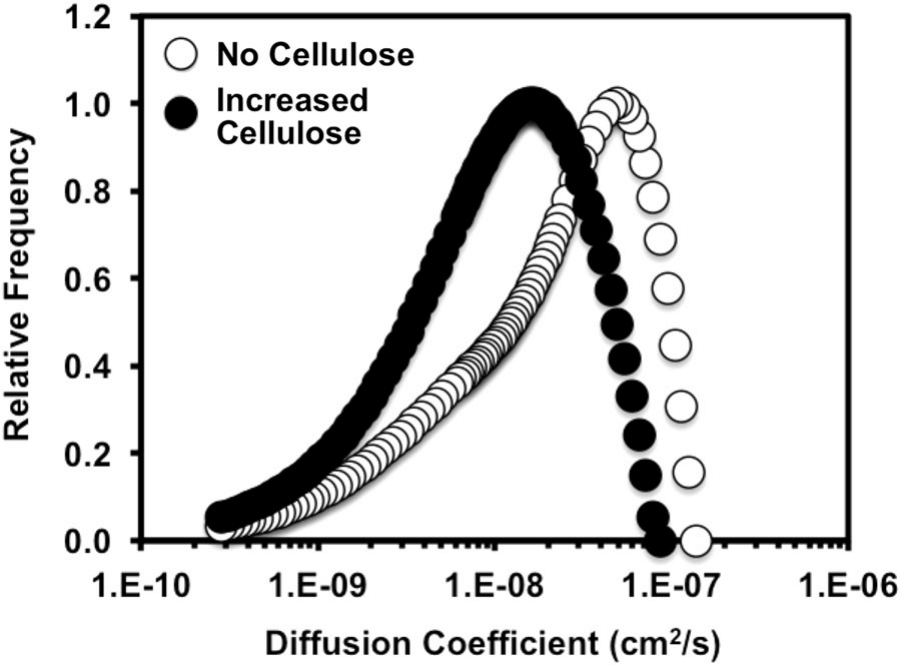
Relative frequency of diffusion coefficients of extracted EPS (no cellulose and increased cellulose) measured using DLS

Responses to nutrient availability join the influences of shear forces as the two strongest external influences on biofilm morphology (Flemming et al., 1997) (Fig. 5). The web-like structure and high networking affinity of cellulose^13,14^ may increase the density of the EPS, and decrease the diffusivity of nutrients and waste through the biofilm. We decided to investigate this influence by dynamic light scattering (DLS) of the EPS solution. DLS analysis is based on fluctuations (constructive or destructive interference by the surrounding colloids) in a laser light scattering intensity of surrounding colloids. Dynamic information of the macromolecules and colloids in the EPS solution can be derived from an autocorrelation of the intensity trace recorded during the DLS measurement, which monitors the Brownian motion of the individual macromolecules. At short time delays, correlation function of the intensity trace recorded during the experiment is high, because the particles do not have a chance to move to a great extent, as the time delays become longer, the correlation decays exponentially.^34^ This exponential decay is related to the motion of the macromolecules and colloids in the EPS solutions, and specifically to the distribution of the diffusion coefficients of the entire EPS macromolecules and colloids. The average diffusion coefficient in the EPS solution of the no cellulose strain, *D*(no cellulose) = 3.6 × 10^−8^ ± 2.6 × 10^−9^ (SE) cm^2^/s, was found to be almost twice as high as in the increased cellulose strain, *D*(increased cellulose) = 1.6 × 10^−8^ ± 9.8 × 10^−10^ (SE) cm^2^/s. The magnitude and significance of this difference (*p* > 0.0001) suggests that increasing cellulose may have tremendous influence on the transportation colloids and macromolecules in the EPS matrix of the biofilm. Morphologically, these reductions in rates of diffusion may explain why the characteristic colony size decreases as cellulose expression increases.

Figure 2 shows a higher ratio of live to dead cells in the biofilm with no cellulose compared to the two other biofilms that do contain cellulose. This live/dead staining data corroborate with our supposition of lower diffusion rates that are attributed to cellulose expression and the associated losses in cell viability. Another influence on the ratio of live/dead cells could be related to the increased concentrations of eDNA, correlated to cellulose production. The eDNA in biofilms can result from active secretion or controlled cell lysis^35^ and controlled lysis could contribute to a lower live/dead ratio. Hence, further research is needed to understand the impacts of cellulose on biofilm fitness.

### Cellulose expression is mediating biofilm elasticity

**Fig. 6:**
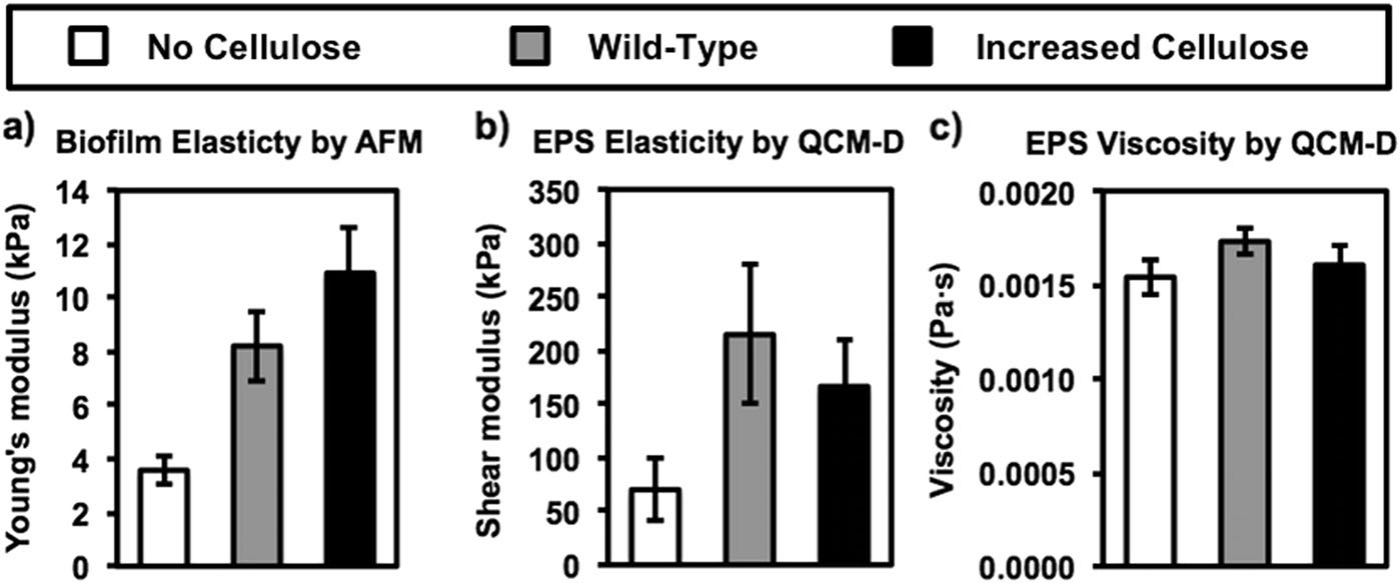
Biofilm elasticity measured by AFM (**a**), and EPS shear modulus (**b**) and shear viscosity (**c**) measured by QCM-D for no cellulose, wild-type, and increased cellulose *V. fischeri* strains. Error bars represent 1 standard error

The effect of cellulose on biofilm elasticity was elucidated using atomic force microscope (AFM) nanoindentation performed on biofilms, and QCM-D viscoelastic modeling^36^ providing adsorption of extracted EPS layer, for each cellulose variant. In the AFM nanoindentation technique, a slow indentation of the surface of the biofilm (1 µm/s) was performed using a 5-µm glass sphere attached to a cantilever with spring constant of 0.06 N/m. The amount of force exerted on the biofilm by the probe (~10 nN) is minimized to ensure an elastic and not a plastic response from the biofilm. By monitoring the bending of the cantilever with respect to the position of the probe in the biofilm, the Young’s modulus of the biofilm was calculated using established models.^37^ A significant difference of the Young’s modulus was observed between the three biofilms, differentially expressing cellulose (Fig. 6a). The wild-type biofilm displayed significantly higher (*p* > 0.001) Young’s modulus of 8.2 ± 1.3 (SE) kPa than the no cellulose strain Young’s modulus of 3.6 ± 1.7 (SE) kPa. The Young’s modulus of the increased cellulose strain was the highest of the three strains, 10.9 ± 0.52 (SE) kPa, not significantly higher than the wild-type (*p* = 0.12) but significantly higher than the no cellulose strain (*p* > 0.001). These values of Young’s moduli are comparable to values obtained in biofilms by previous studies.^38,39^

The shear modulus and the shear viscosity of EPS, extracted from biofilms of each cellulose-expression mutant, were measured using QCM-D to verify the findings observed with the AFM (Figs. 6b, c). In addition to the measurements of a bound mass of EPS, which is deduced from changes in the resonance frequency, ∆*f*, of the piezoelectric sensor (values shown for the end of EPS adsorption step in Figs. 4d, e), the QCM-D technique also provides information on the rheology of biomolecular layers via changes in the damping, ∆*D*, of the crystal.^40,41^ Using the Voigt-based viscoelastic model,^36^ key properties of the biopolymers and macromolecules including elastic shear modulus, shear viscosity, and layer thickness can be calculated.^42,43^ Various types of biopolymers, or EPS, have been reported as exhibiting different type of interactions with the substratum, which correlated to their viscoelastic properties determined in the QCM-D.^44,45^ The shear modulus and shear viscosity can then be calculated using changes in frequency, ∆*f*, and dissipation factors, ∆*D*, at different overtones, using the Voigt model. This analysis yielded shear modulus values of 70 ± 29, 215 ± 65, and 167 ± 44 (SE) kPa for no cellulose, wild-type, and increased cellulose strains, respectively (Fig. 6b). Statistically different shear modulus were observed when comparing EPS that did not contain cellulose to the EPS from the two cellulose-containing strains (*p* > 0.05). The difference in shear modulus between the wild-type and increased cellulose strains is not statistically significant (*p* ≥ 0.1). One possible explanation for the difference in the elasticity analyzed with the QCM-D and the AFM is that the AFM is intrinsically bulk measurement and the QCM-D measurement is dominated by the attached EPS layer. Other reason is the difference in the rheological spectra of the EPS analyzed with AFM vs. QCM-D, which links between the vibration frequency and the viscoelastic properties. In addition, the process of extracting and re-depositing the EPS allows us to examine EPS without differences and heterogeneities of the biofilm structure; however, the difference may contribute to explaining why the QCM-D experiments did not yield exactly the same statistically significant difference with cellulose expression observed in the relatively direct AFM experiments.

The QCM-D modeling of EPS shear viscosity yielded near identical results (*p* ≥ 0.25) for each cellulose expressions strain, with 1.5 × 10^−3^ ± 2.9 × 10^−4^, 1.7 × 10^−3^ ± 6.5 × 10^−4^, and 1.6 × 10^−3^ ± 4.4 × 10^−4^ (SE) Pa s, for no cellulose, wild-type, and increased cellulose strains, respectively (Fig. 6c). Though the modeling of shear viscosity exhibits the same limitations as the modeling of shear modulus, the similar viscosities reported for each strain imply that cellulose expression does not affect the viscous behavior of these biofilms. This strengthens the claim that the observed differences in AFM Young’s modulus are indeed caused by elastic responses that the AFM identifies, and not from differences in plastic deformations. Therefore, it is likely the elasticity of the biofilms that is different at different levels of cellulose expression. This is the first study that quantitatively evaluates Young’s modulus in relation to cellulose expression in biofilms. The correlation between increasing Young’s modulus and increasing cellulose expression is consistent with our expectations, based on the *β*-glycosidic backbone and the dense network of hydrogen bonding present in cellulose.^13,14^ This correlation is also consistent with qualitative observations performed on *Salmonella typhimurium* in which an isogenic strain not producing cellulose displayed more elastic behavior, relative to the wild-type, which does produce cellulose.^11,20^ In addition to the expected effects based on the physical and chemical roles of cellulose as extracellular scaffold, we suggest that biofilms elasticity may also be affected by the presence of eDNA, mediated by cellulose expression as shown by the eDNA analysis in the EPS of the cellulose variants. eDNA was reported to play an important role in the viscoelastic relaxation of biofilms by a thorough mechanical deformation and relaxation analysis of 104 different biofilms of *Staphylococcus aureus*, *Staphylococcus epidermidis*, *Streptococcus mutans*, and *Pseudomonas aeruginosa*.^46^ Peterson et al. showed the importance of eDNA among other EPS components, using principal component analysis, possessing eDNA as a unique principal component with a time relaxation constant in the range of 10–25 s.^46^ In this study, both cellulose and eDNA seems to affect the elastic properties of the EPS layer adsorbed to the QCM-D sensor. Corroborating with the QCM-D results, elevation in Young’s modulus were detected directly by nanoindentation with AFM. While we did not isolate the relative contribution of each of these components, we can speculate that possible weak interactions between eDNA and cellulose in the extracellular matrix may enhance biofilm rigidity (by means of hydrogen bonds, van der Waals forces, and electrostatic effects). Such interactions of eDNA with extracellular polysaccharides and their effects on biofilm mechanical properties are definitely important subject of future studies.

The most direct impacts of elasticity on biofilm development and morphology may be related to altering how developed biofilms respond to shear forces. Robust models have been developed to describe the slipping, peeling, and removal of coherent bodies attached to surfaces by viscoelastic adhesive layers.^47–51^ The most applicable finding of these studies when investigating biofilms under shear flow is the greater resistance to peeling exhibited by materials with low elasticity. Peeling is initiated by a break in the upstream adhesion between the body and the surface, which requires the edge of the body to bend back into the shear flow, away from the surface. This peeling mechanism significantly reduces the total force required to remove the adhesive body from the surface, and less elastic materials will be more resistant to this bending and the initiation of the peel. Probably, in the case of cellulose expression biofilms, the reduced biofilm elasticity, as cellulose expression increases, results in greater resistance to biofilm removal from the surface, increasing the number of colonies and the total biovolume. The significance of elasticity in the biofilm resistance to shear is also supported by our QCM-D experiments, showing that cellulose does not affect the affinity of EPS to adhere on polyamide (Figs. 4d, e).

## Concluding remarks

The expression of cellulose in *V. fischeri* biofilms has significant impacts on the mechanical properties, and, in turn, on the morphological characteristics of the biofilm. Increasing cellulose expression elevated the extent of eDNA in the biofilms, increased the Young’s modulus of the biofilm, and reduced the size of colonies. Consequently, increasing cellulose expression elevated the total volume of *V. fischeri* biofilms. The associated cellulose variants were investigated for differences in cellular deposition, cellular hydrophobicity, cellular surface charge, adherence of their self-produced EPS matrix, and growth rates. However, these characteristics were not found to influence significantly biofilm morphology. The observed differences in morphology are suggested to arise from the higher Young’s modulus and the reduced diffusion rates mediated by cellulose and eDNA in the extracellular scaffold.

## Materials and methods

### Bacterial strain selection and cultivation

Three *V. fischeri* bacterial strains have been analyzed: a wild-type (KV4674), an isogenic mutant Δ*binA*, which overexpresses cellulose (KV4131), and an isogenic mutant Δ*bcsA*, which does not express cellulose (KV5366).^52,53^ We note that *V. fischeri* has been reclassified as *Aliivibrio fischeri*.^54^ Each strain was grown on a LBS agar and broth at 25 °C.^55^ All experiments were conducted with stationary phase bacteria, prepared by inoculating an LBS overnight culture in fresh LBS media at a ratio of 1:1000 and incubating for approximately 16 h at 25 °C and 150 rpm. For the bacterial deposition assay, these three cellulose expression variants were labeled with a plasmid (pESY37) containing a *gfp* gene,^56^ for real-time observation of the cell attachment to a polypropylene membrane.

### Biofilm growth and harvesting

Biofilms grown for imaging with CLSM were prepared using polypropylene membranes as a substratum mounted in a continuously fed flow cell at 25 °C. The hydrodynamics of this system have been previously described in greater detail.^26,27^ Schematic figure of the flow cell is presented in the Supplementary information (Fig. S1). In brief, an average cross flow velocity of 0.44 cm/min was achieved with a corresponding wall shear rate of ~8.4 per minute. The flow cell was sterilized with 70 % ethanol for 2 h, washed with autoclaved DI water, and then inoculated for 30 min with a stationary phase culture of bacteria at a flow rate of 2 ml/min. The feed was then switched to LBS media, also at 2 ml/min for 24 h. After 24 h, the fouled polypropylene membrane was removed from the flow cell, gently washed by submerging in 0.34 M NaCl, sampled and immediately processed for CLSM. The procedure for biofilm growth for AFM analysis is identical to that for CLSM, except for the glass slides being used as a growth substratum, since a rigid substratum was required for the AFM measurements.

### Confocal laser scanning microscopy

SYTO-9 and PI stains (Invitrogen Eugen, Oregon, USA) were used to stain live and dead bacteria, respectively, using established protocols.^57^ Briefly, 1.5 μl of a 30 mM PI concentration and 1.5 μl of 3.34 mM SYTO-9 were inserted into 1 ml of 100 mM NaCl. The sample membrane was then covered with the staining mix by pipetting, incubated for 10 min in the dark and gently washed three times with 100 mM NaCl. Two independent biofilm growth experiments were carried out for each of the cellulose variants. The developed biofilms were then visualized using a CLSM (Zeiss-Meta 510, Zeiss, Oberkochen, Germany), with images collected from eight positions on each membrane (representative images are shown in Supplementary Figs. S2–S4). Image processing and determination of specific biovolume values (μm^3^/μm^2^) were conducted using IMARIS 3D software (Bitplane, Zurich, Switzerland).

### Bacterial deposition

Rates of bacterial deposition were directly measured in duplicate by microscopy using a parallel plate flow cell according to our previous study.^26^ Briefly, polypropylene membranes (0.2 µm, Pall life Sciences, Port Washington, NY, USA, P/N 66557) were mounted to a glass slide inside a flow cell (Biosurface Technologies Co. Bozeman, MT, USA, model FC81) and fed with stationary phase bacteria. The stationary phase bacteria were diluted with bacteria-free LBS to an optical density at 600 nm (OD_600 nm_) of 0.01 (concentration of 5 × 10^−6^ ± 1 × 10^−6^ cells/ml) and peristaltically pumped into the flow cell at a flow rate of 2 ml/min, which corresponds to an average velocity of 9.6 cm/min and a wall shear rate of 360 per minute. The significantly higher shear rate value applied for the bacterial deposition experiments, compared to the biofilm growth conditions, ensured statistically significant elevation in cell counts with time. At lower shear rates, closer to the hydrodynamic condition applied for biofilm growth, the changes in cell counts on the surface were marginal. At these conditions, the Smoluchowski–Levich approximation of the two-dimensional convective-diffusion equation is commonly used to predict mass transport of bacterial cells to the surface, in which the flux of bacteria to the surface is elevated with shear rate^58^ at values smaller than ~200 per second.^59^ The deposition was enumerated using an optical microscope (Zeiss, ZX10) under 40X magnification at 5 time points approximately 25 min apart. The cell deposition flux is reported as the observed deposition rate of bacteria normalized by the camera viewing area. Counts at the final time point ranged from 127 to 334 cells per field of area 0.035 mm^2^ and there was no evidence of accumulation affecting deposition.

### Surface analysis of bacteria

Relative hydrophobicity of each bacterial strain was determined using a microbial adherence to hydrocarbon test.^60,61^ In brief, triplicate 4-ml suspensions of each stationary-phase bacteria were adjusted to an OD (600 nm) of 0.3 and transferred into a test tube mounted with 1 ml of *n*-dodecane hydrocarbon (Biolab LTD-chemicals, Jerusalem, Israel). The tubes containing the bacteria were incubated at 25 °C under gentle shaking for 10 min, followed by 2 min of vortexing and 30 min of rest, to allow for phase separation. The pp% was determined to be the difference between final and initial OD, divided by the initial OD.

Triplicate measurements of bacterial electrophoretic mobility were performed (with 10 measurements per culture) using a zeta potential analyzer (ZetaPlus 1994, Brookhaven Instruments Co., Holtsville, NY, USA) according to de Kerchove and Elimelech.^62^ All cultures were washed and then diluted in 150 mM NaCl to an OD (600 nm) of 0.1 prior to analysis at 25 °C. Electrophoretic mobility measurements were converted into zeta potentials by using the Smoluchowski equation. This equation was applicable because of the relatively large cell size and high ionic strength tested.^63^

### EPS harvesting and extraction

In order to produce relatively large amount of EPS, a substratum with relatively high surface area supporting large amount of biomass was used. Biofilms of each bacterial strain were grown in pure culture on continuous-flow vertical columns (1-inch diameter, 100 ml total volume) filled with glass beads (425–600 μm, Sigma Aldrich Israel, cat# G9268). Columns were fed at the bottom with a peristaltic pump and wasted from the top. The columns, beads, fittings, and tubing were sterilized by flowing 70 % ethanol through the system for 2 h, followed by autoclaved DI water for 30 min. An overnight culture of the bacterial strain of interest was then pumped into the column for 30 min at a flow rate of 2 ml/min. The column was then fed LBS media for 48 h also at 2 ml/min. Since the hydrodynamics and nutrient distribution in this system likely vary compared to the biofilms developed in the parallel flow cell and affect the EPS composition, we aimed on a limited quantitative comparison between the different types of EPS. In addition, the genetically different cellulose expression will likely have a greater impact on such a comparison than hydrodynamics.

After the biofilm growth phase, each column was disassembled, residual liquid media was wasted, and the biofilm-covered beads collected and gently washed two times with 0.145 M NaCl. Beads were immersed in 50 ml of 0.145 M NaCl and 0.3 ml of 36 % formaldehyde, and incubated for 1 h at 4 °C under gentle shaking. Next, 20 ml of 2 M NaOH was added and beads were returned to the 4 °C shaking incubator for an additional 3 h. The beads were wasted, and the liquid portion was centrifuged for 30 min at 4 °C. Supernatant was filtered through 0.22 μm hydrophilic filters (Millipore, Billerica, MA, USA) and dialyzed with a 3500 Dalton membrane (Spectrum Laboratories Inc., Rancho Dominguez, TX, USA) against DI water until the conductivity of the solution dropped below 1 μS/cm. These conditions provided reasonable comparative measure for the amount of cellulose in the EPS, in which the degree of polymerization is below ~390.^25^ Stocks of EPS were stored at −20 °C. The TOC in the EPS was measured using an Apollo 9000 combustion TOC analyzer (Teledyne Tekmar, Mason, OH, USA). EPS extracted from the differentially expressed cellulose production of biofilms were diluted to 175 mg/l TOC using DI water. As the EPS extraction method used in this study may lead to cell destruction and intracellular contaminates, cell counts of the pellets before and after the EPS extraction revealed no significant difference and therefore insignificant intracellular contamination (results not shown). The concentrations of eDNA were then measured using a Quant-iT PicoGreen dsDNA Assay (Life Technologies, Thermo Fischer Scientific, Waltham, MA, USA). EPS samples were diluted 100 fold in TE buffer, incubated with the PicoGreen reagent, and analyzed against a standard curve using a fluorescent plate reader (Synergy H4 with Gen 5 software, Bioteck Inc., Winooski, Vermont, USA).

### Relative quantification of cellulose in the EPS solutions

A relative quantification of cellulose in the EPS was done in triplicate by the calcofluor-binding assay. All EPS solutions (5 ml) were diluted to a similar concentration of 63 mg/l as TOC and incubated in dark for 24 h with calcofluor White Stain (Sigma-Aldrich, Israel, cat# 18909) diluted to 50 mg/l in 10-mM phosphate buffer solution (pH 7.2). Thereafter, each of the EPS solution (2 ml) was filtered through an ultrafiltration membrane (5 KDa molecular weight cut-off (MWCO)) (Microdyn-Nadir, Wiesbaden, Germany) and unbounded calcofluor stain was washed two times (4 ml) with 10-mM phosphate buffer solution. Then, membranes with adsorbed EPS were cut to pieces of ~2 × 2 mm and vortexed for 15 min in 5 ml of the phosphate solution for removal of the EPS adsorbed to the membrane. Finally, EPS solutions were diluted again four times and three equal volumes (300 μl) were added from each replicate to a 96-well microtiter plate. The plate was incubated in an auto microplate reader (Infinite M200, Tecan) with the monochromators set for excitation at 355 nm and emission at 434 nm. Controls of the EPS solutions without calcofluor stain had similar emission at 434 nm as the 10-mM phosphate buffer blank solution, which was subtracted from the fluorescence unit readings of the different EPS solutions.

### EPS adsorption assay using QCM-D

The EPS extracted from the continuous-flow vertical columns filled with glass beads was used for adsorption assay in the QCM-D system. EPS adsorption to polyamide-coated quartz sensors (Q-Sense AB, Gothenburg, Sweden) was measured in duplicate using an E4 QCM-D system (Q-Sense AB, Gothenburg, Sweden). For each experiment, the solutions were injected sequentially using a digital peristaltic pump (IsmaTec, Switzerland) at a flow rate of 150 µl/min in the following order: (1) DDW for 20 min; (2) 0.34 M NaCl (background solution) for 30 min; (3) background solution supplemented with 12.5 mg/l (TOC) EPS for 60 min; (4) EPS-free background solution for 45 min; (5) DDW for 45 min. Viscoelastic properties (shear modulus and shear viscosity) of the EPS layers were calculated using the Voigt model^36^ implemented in Q-Tools software (Q-Sense AB, Gothenburg, Sweden). The input raw data for Voigt modeling included changes in frequency and dissipation factors during the deposition of EPS onto QCM-D sensors for at least two overtones.

### Exponential growth rate measurement

Six different stationary phase cultures (5 ml) of each cellulose expression strain were reinoculated at a ratio of 1:1000 in LBS (5 ml) and grown 8 h to reach log phase (Supplementary information, Figs. S11–S13). Each of these three cultures was then reinoculated at a ratio of 1:1000 in LBS (100 ml) in three separate Erlenmeyer flasks for each strain. The OD (600 nm) was measured for each culture at time zero and then at 10 times over the following 6 h. Growth rates were obtained from linear regression of OD (600 nm) values in the exponential growth region of the curve, where the natural log of OD (600 nm) plots linearly vs. time.

### Diffusion coefficient distribution measured by DLS

Distribution of the diffusion coefficients were measured in triplicate samples of extracted EPS from each variant of the cellulose expression biofilm using a DLS system (ALV/CGS-3 Goniometer, ALV/LSE-5004 Correlator, ALV/GmbH, Langen, Germany). EPS samples were adjusted to 100 mg/l as TOC in DI water and filtered through 0.22 hydrophilic polyvinylidene fluoride (PVDF) filter (Millipore). Scattering angle was set at 90° for a collection time of 2 min at 25 °C.

### Atomic force microscopy

At least 30 force curves were collected from biofilms of each cellulose expression variant using a Veeco/Bruker AFM (Multimode with IIIa controller, Bruker Corp., Billerica, MA, USA) in 0.34 M NaCl background solution, using a 5-µm glass bead probe attached to a 0.06 N/m cantilever (Novascan Technologies Inc., Ames, IA, USA) with an approach and retraction speed of 1 µm/s (representative force curves are shown in the Supplementary information Figs. S5–S7). Young’s modulus was determined using the Hertzian model,^37^ assuming a Poisson ratio of 0.5. Visualization and calculations were performed using NanoScope Analysis software (Version 1.40, Bruker Corp., Billerica, MA, USA).

## Electronic supplementary material

Supplementary Information
