# Supplementary material for: Cellulose effects on morphology and elasticity of Vibrio fischeri biofilms
Source: NPJ Biofilms Microbiomes. 2016 Nov 3;2:1. doi: 10.1038/s41522-016-0001-2 (PMC5460256; doi:10.1038/s41522-016-0001-2)
Supplement: Supplementary file 1 — Supplementary Information [file 41522_2016_1_MOESM1_ESM.docx]

Cellulose effects on biofilm morphology and elasticity of *Vibrio fischeri* biofilms

**Supplementary Information**

Ziemba C., Shabtai Y., Piatkovsky M., and Herzberg M.*

The Blaustein Institutes of Desert Research, Zuckerberg Institute for Water Research, Ben-Gurion University of the Negev, Sede Boqer Campus 84990, Israel.

* Corresponding author phone: 972-8-6563520 e-mail: herzberg@bgu.ac.il

**Figure S-1:** The multi-channel flow cell for microbial biofilm growth accommodating four glass slides, on which up to eight different membrane stabs may be mounted.


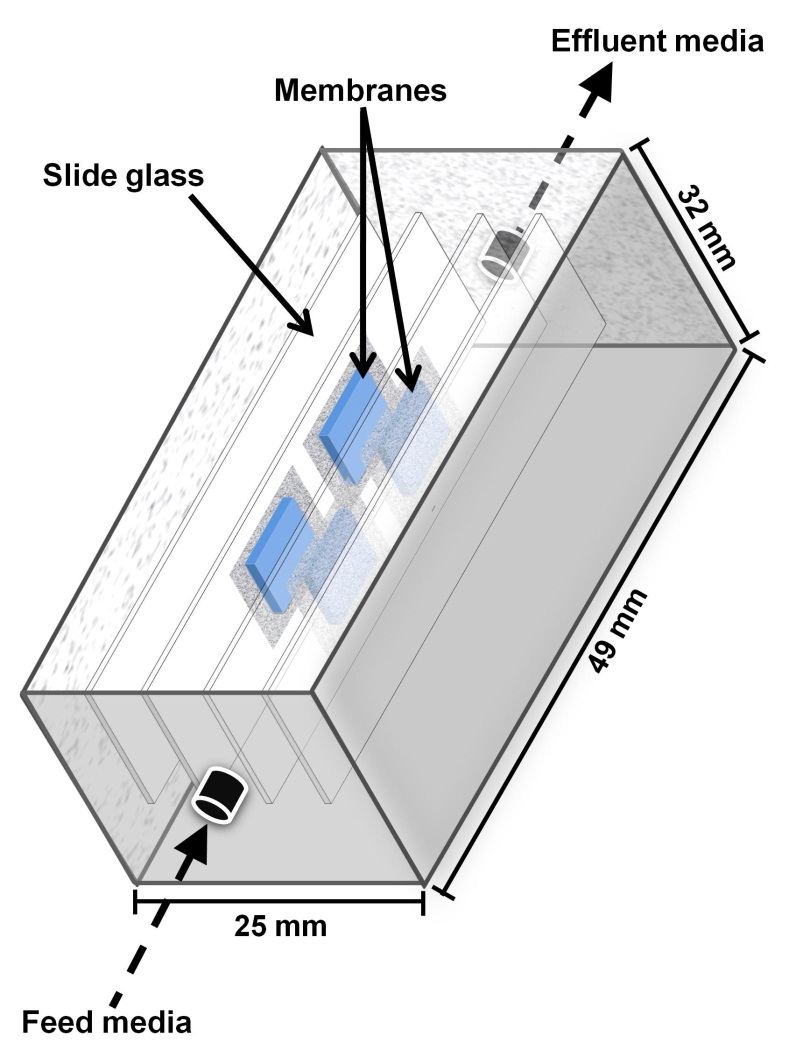


**Figure S-2:** Deletion strain 24-hour CLSM live/dead

a)


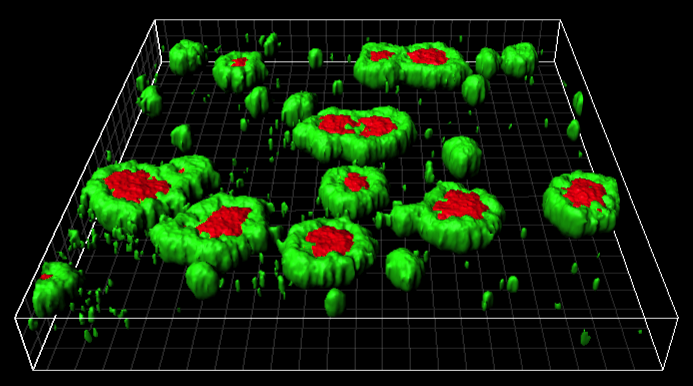


b)


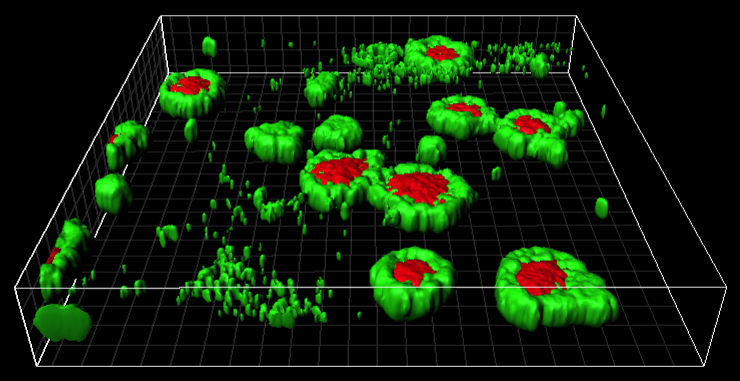


c)


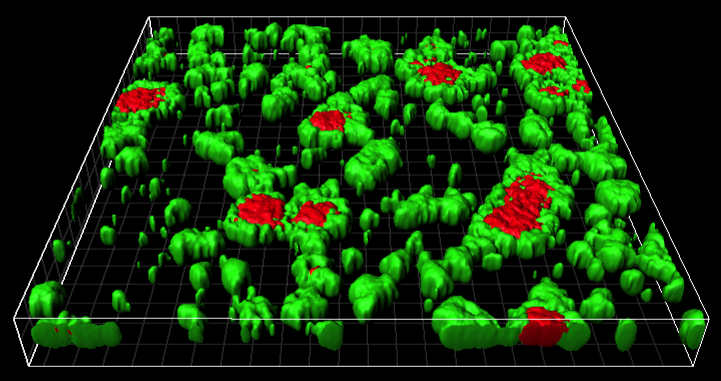


d)


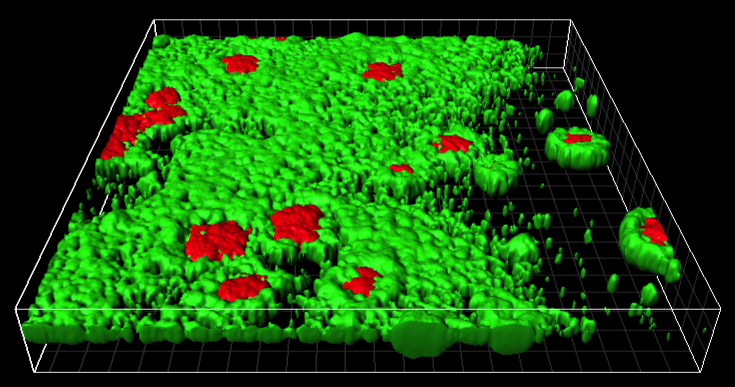


e)


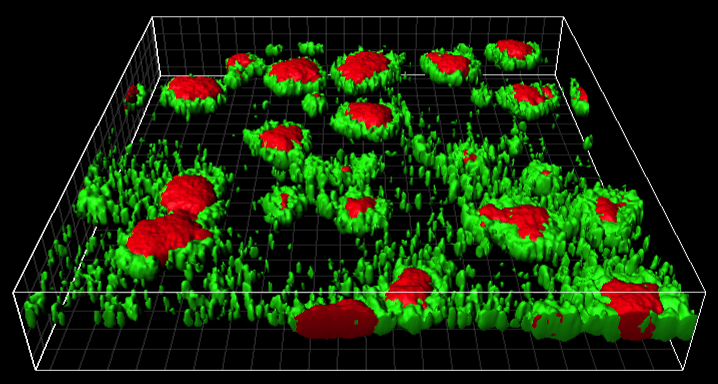


f)


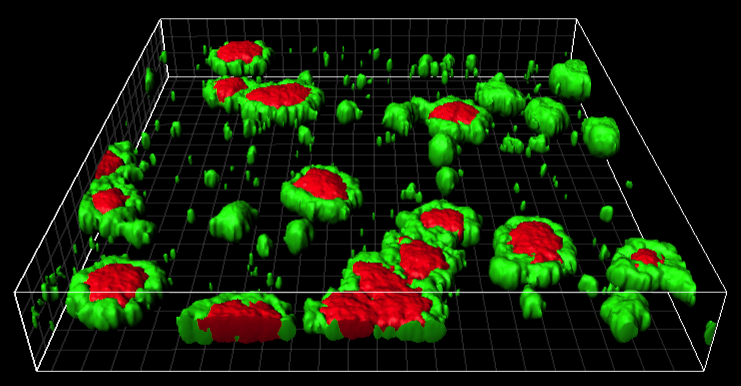


g)


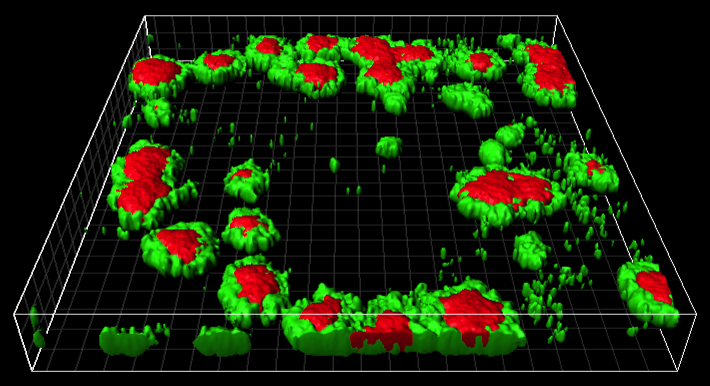


h)


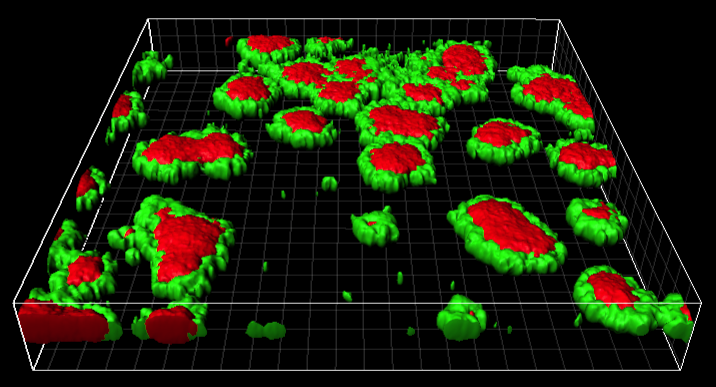


**Figure S-3:** Wild type strain 24-hour CLSM live/dead

a)


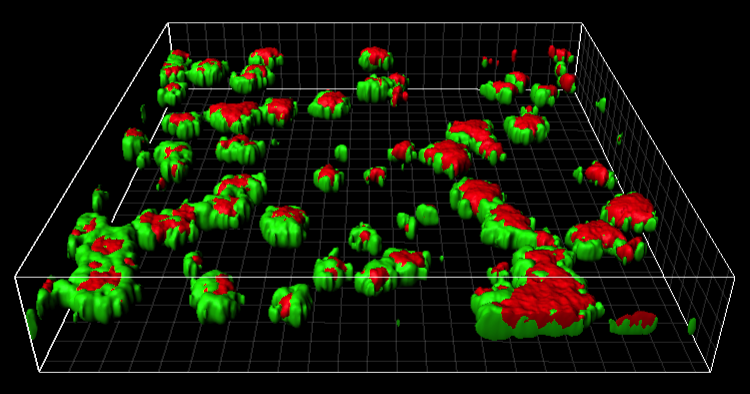


b)


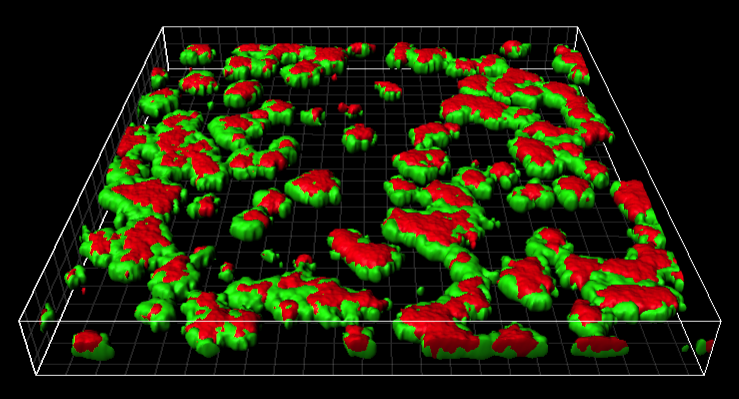


c)


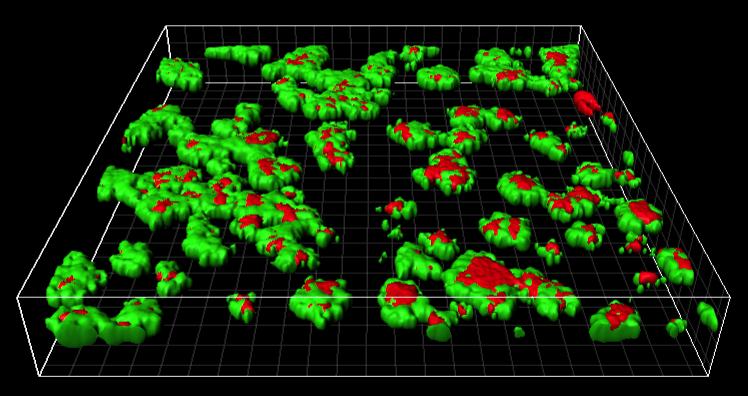


d)


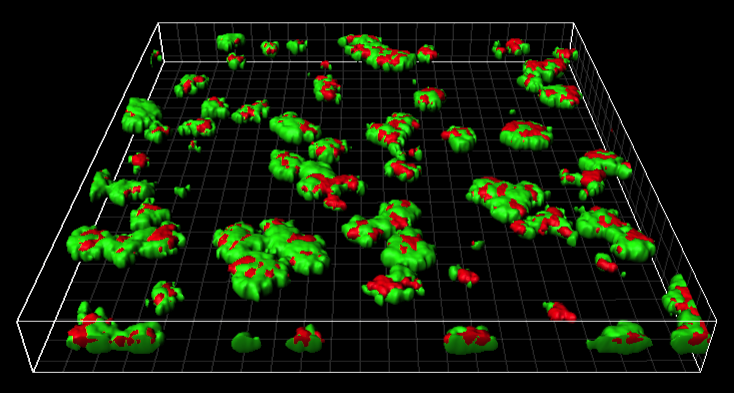


e)


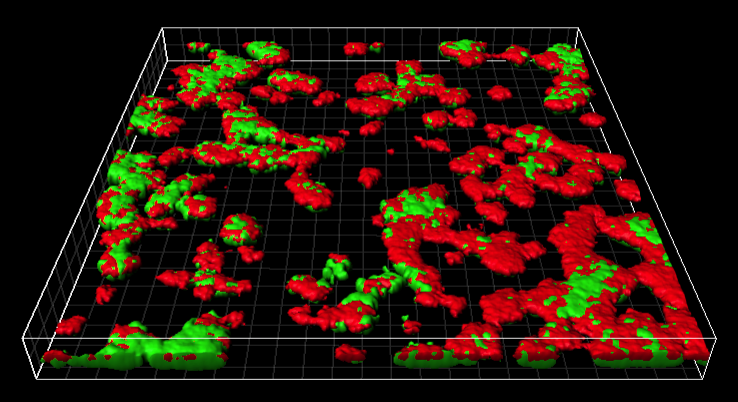


f)


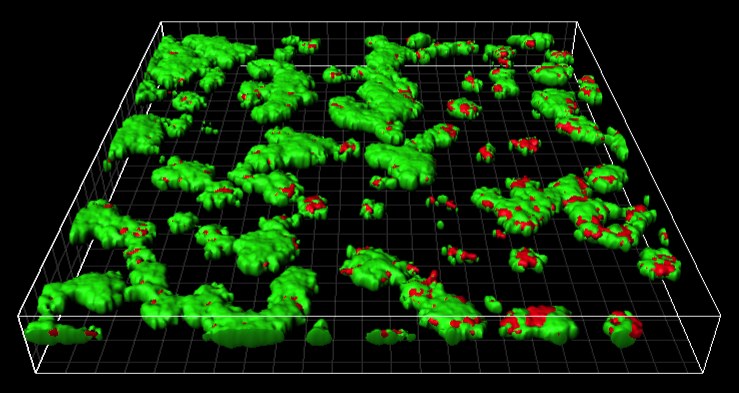


g)


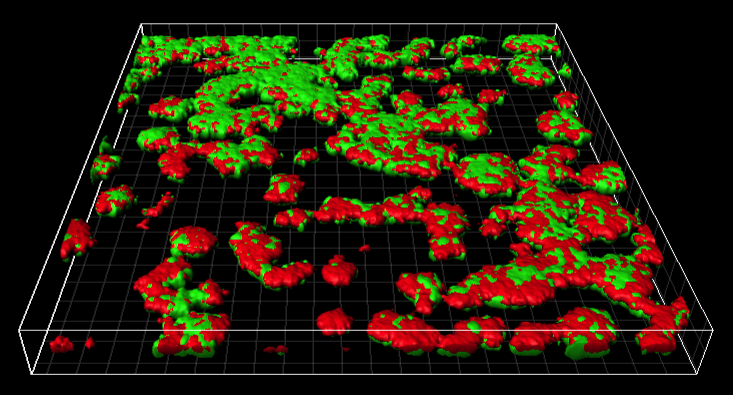


h)


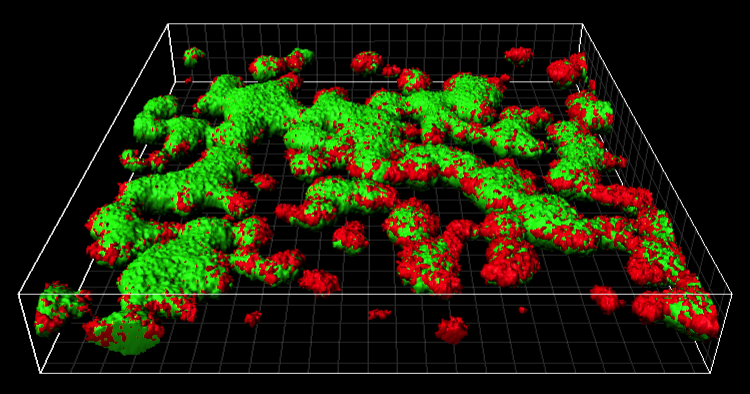


**Figure S-4:** Overexpression strain 24-hour CLSM live/dead

a)


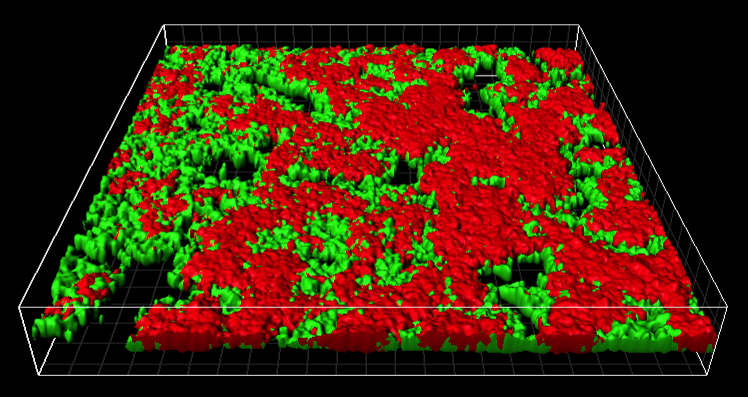


b)


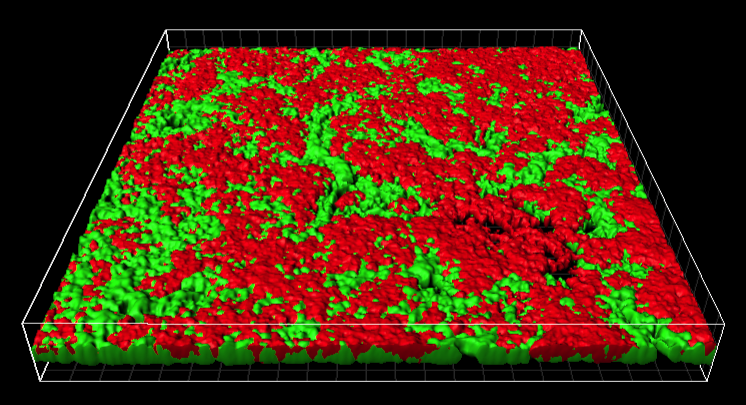


c)


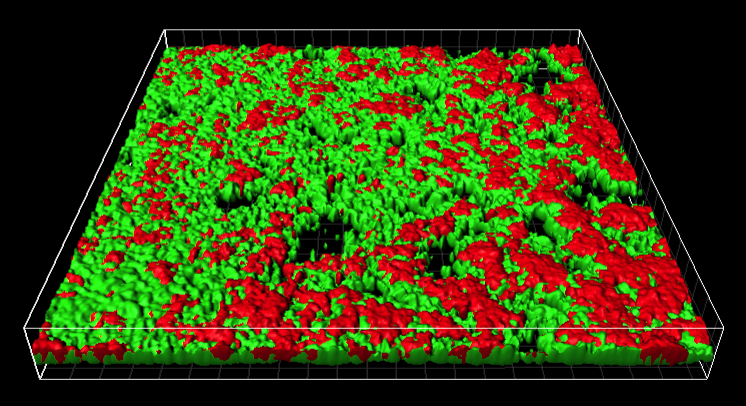


d)


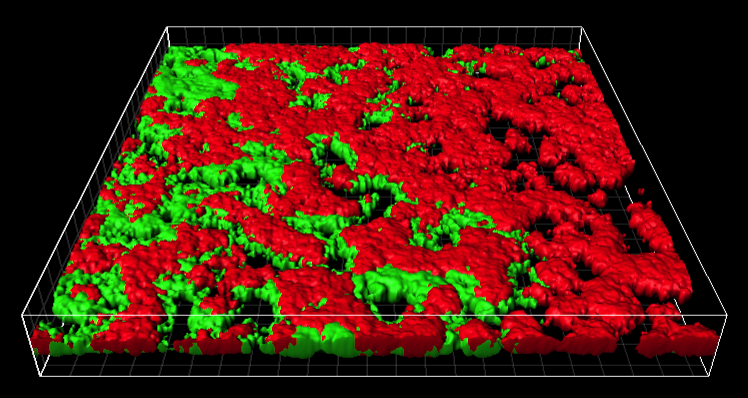


e)


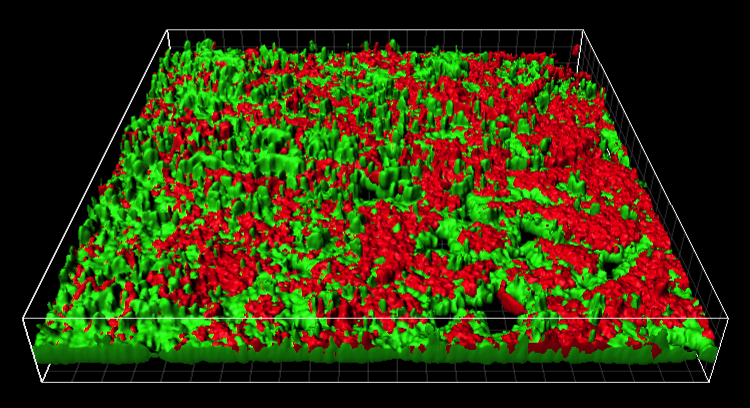


f)


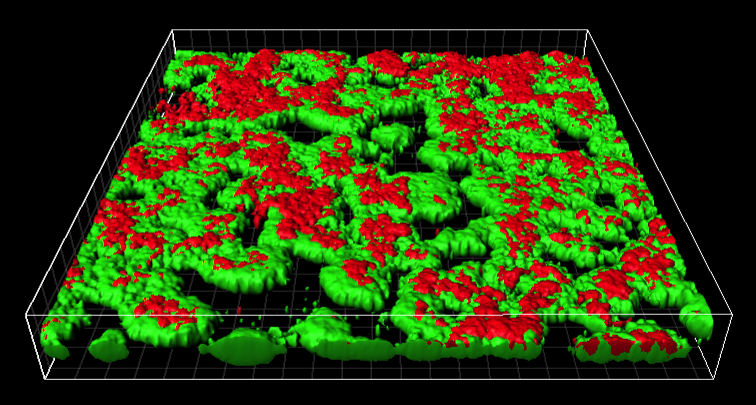


g)


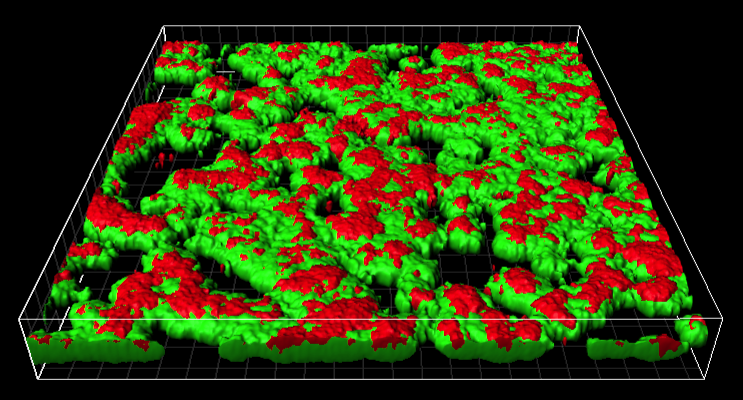


h)


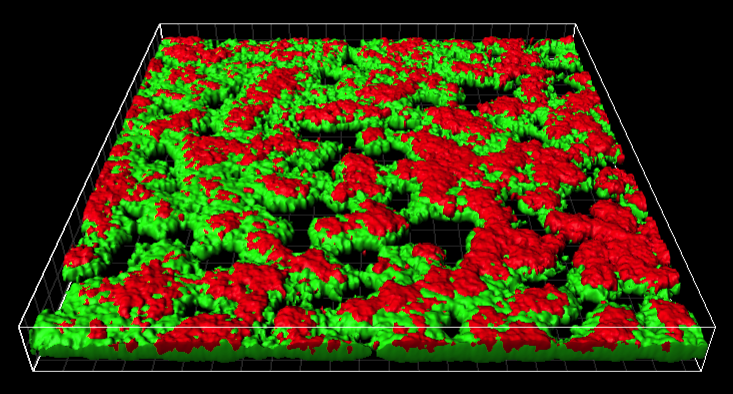


**Figure S-5:** Deletion strain 24-hour AFM force – separation plots

a) 2.15 kPa


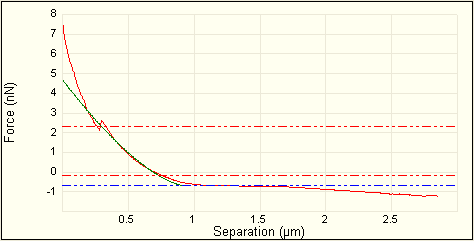


b) 1.83 kPa


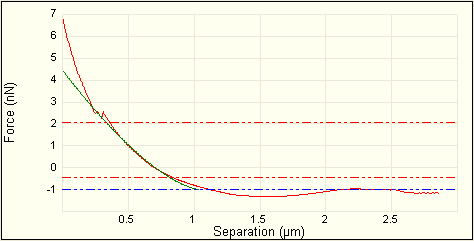


c) 3.25 kPa


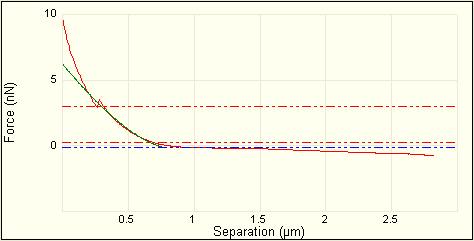


d) 1.99 kPa


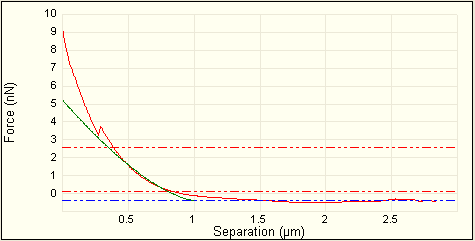


e) 2.75 kPa


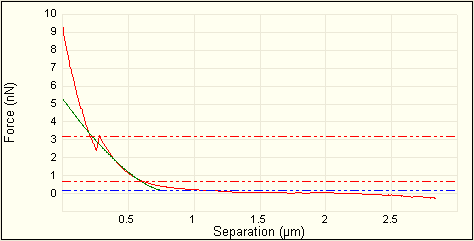


f) 1.76 kPa


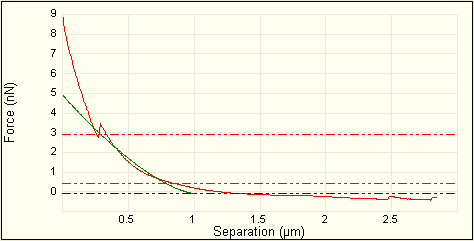


g) 1.16 kPa


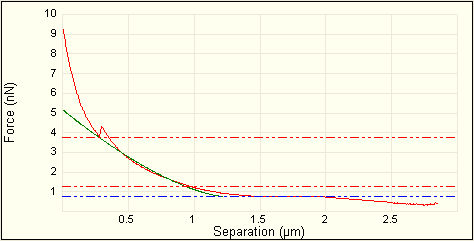


h) 2.71 kPa


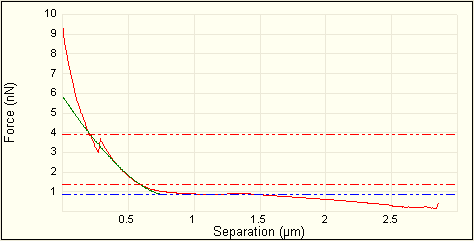


**Figure S-5 continued:** Deletion strain 24-hour AFM force – separation plots

i) 3.54 kPa


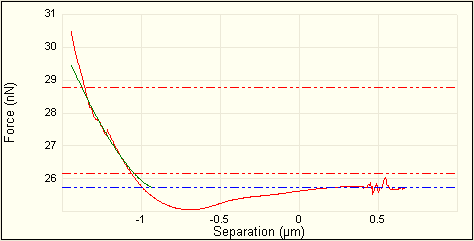
.

j) 4.74 kPa


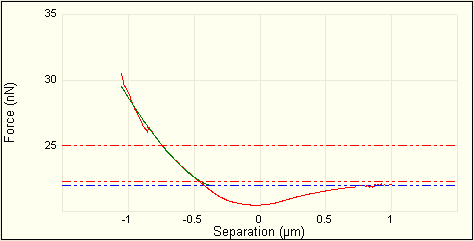


k) 4.52 kPa


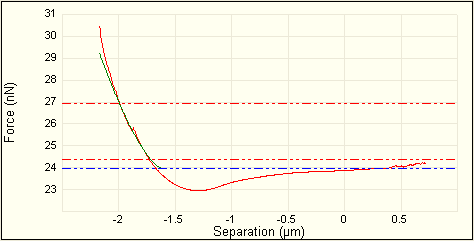


l) 6.13 kPa


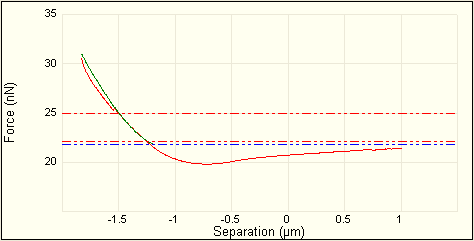


m) 6.22 kPa


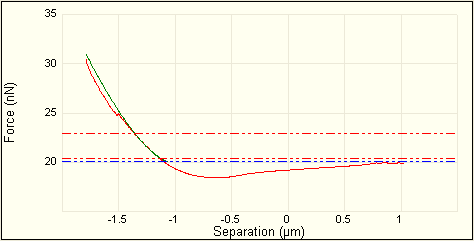


n) 7.35 kPa


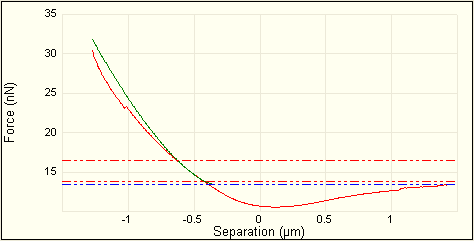


**Figure S-6:** Wild type strain 24-hour AFM force – separation plots

a) 4.29 kPa


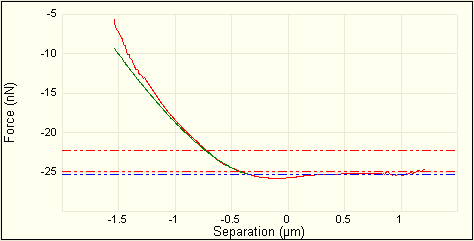


b) 6.57 kPa


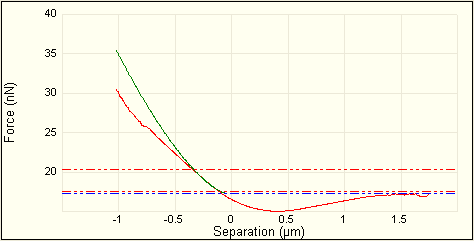


c) 4.32 kPa


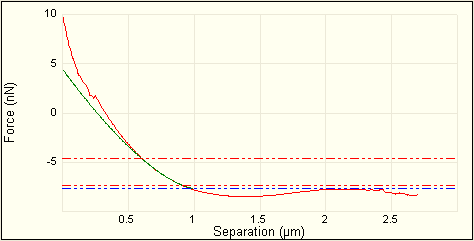


d) 8.54 kPa


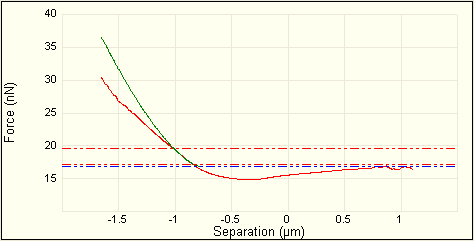


e) 7.58 kPa
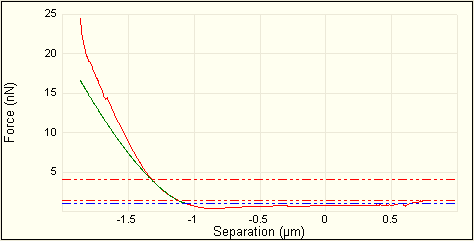


f) 7.14 kPa


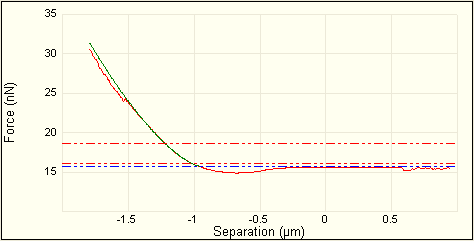


g) 7.91 kPa


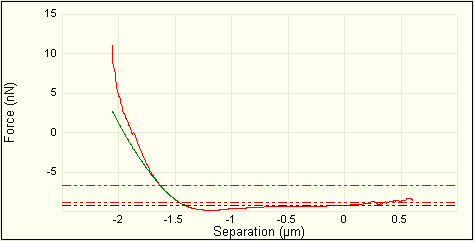


h) 11.1 kPa


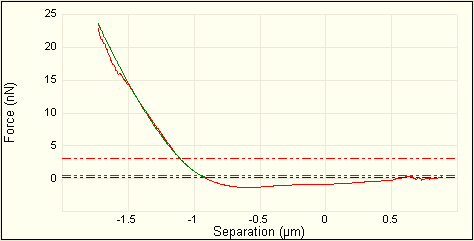


**Figure S-6 continued:** Wild type strain 24-hour AFM force – separation plots

i) 18.4 kPa


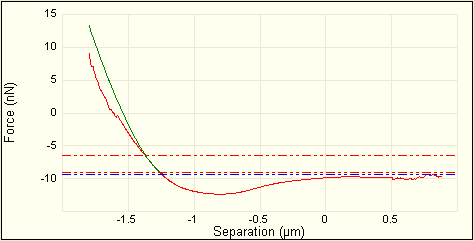


j) 11.8 kPa


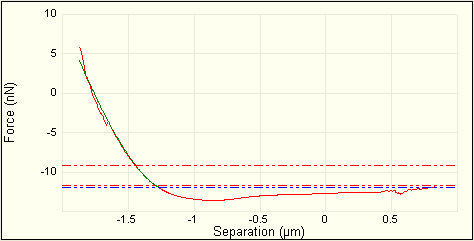


k) 2.72 kPa
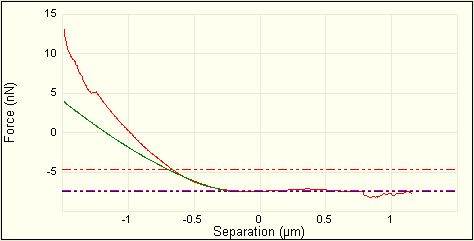


**Figure S-7:** Overexpression strain 24-hour AFM force – separation plots

a) 22.0 kPa


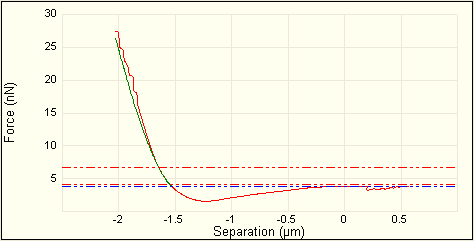


b) 2.18 kPa


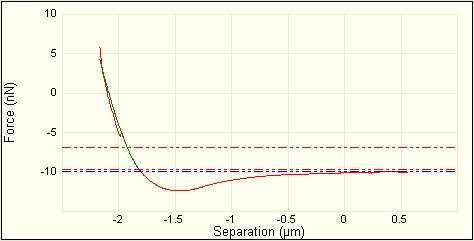


c) 12.1 kPa


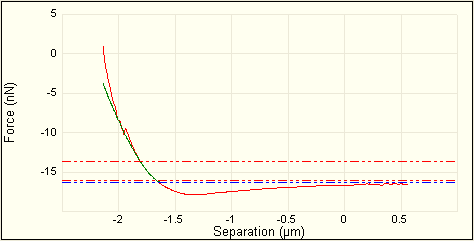


d) 12.0 kPa


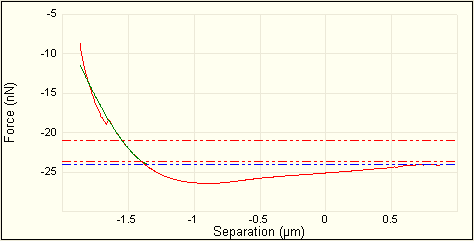


e) 18.7 kPa


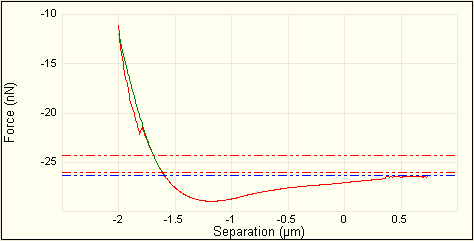


f) 9.57 kPa


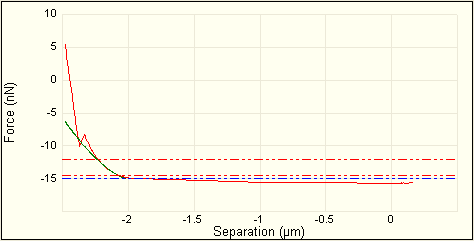


g) 14.3 kPa


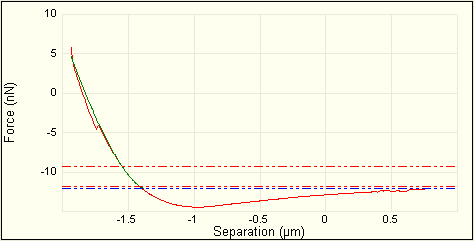


h) 8.34 kPa


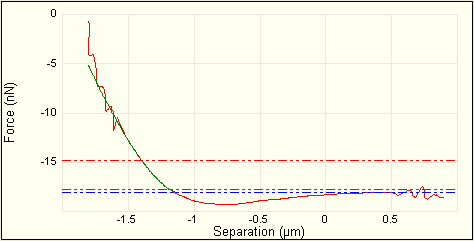


**Figure S-7 Continued:** Overexpression strain 24-hour AFM force – separation plots

i) 9.38 kPa


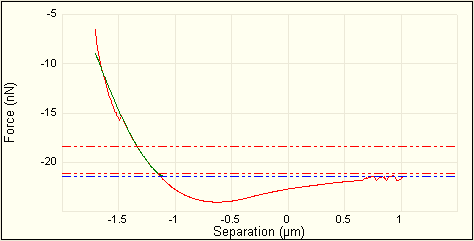


j) 4.65 kPa


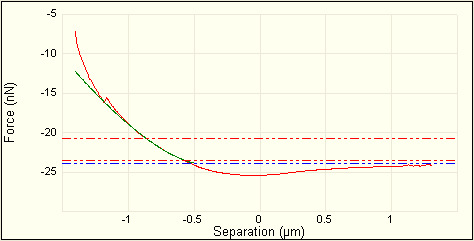


k) 3.55 kPa


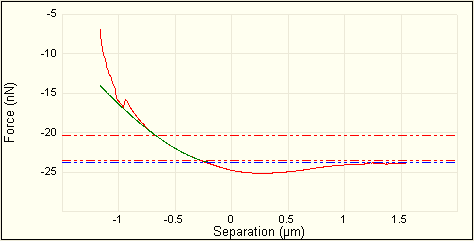


l) 3.99 kPa


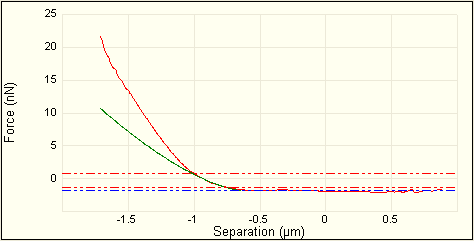


m) 8.80 kPa


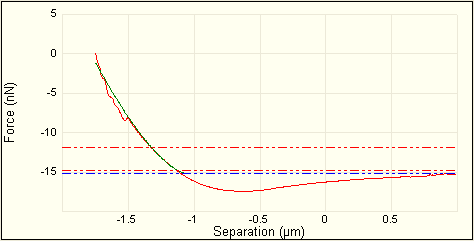


n) 3.64 kPa


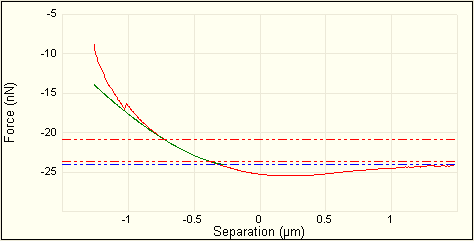


**Figure S-8:** Cellulose deletion bacteria deposition in LBS media on polypropylene membrane (in duplicate)

a) b)

**Figure S-9:** Wild type bacteria deposition in LBS media on polypropylene membrane (in duplicate)

a) b)

**Figure S-10:** Cellulose overexpression bacteria deposition in LBS media on polypropylene membrane (in duplicate)

a) b)

**Figure S- 11:** Cellulose deletion growth in LBS media 25C (composite plot of 6 trials)

**Figure S- 12:** Wild type growth in LBS media 25C (composite plot of 6 trials)

**Figure S- 13:** Cellulose overexpression growth in LBS media 25C (composite plot of 6 trials)
